# Remarkable enhancement of the adsorption and diffusion performance of alkali ions in two-dimensional (2D) transition metal oxide monolayers via Ru-doping

**DOI:** 10.1038/s41598-024-53966-5

**Published:** 2024-02-22

**Authors:** Shubham Sahoo, P. Kumari, Narayan N. Som, S. Kar, Rajeev Ahuja, S. J. Ray

**Affiliations:** 1 Department of Physics, Indian Institute of Technology Patna, Bihta, 801103 India; 2 Laboratory Nanostructures Institute of High Pressure Physics, Polish Academy of Sciences Sokolowska, Warsaw, Poland; 3 Department of Physics, Indian Institute of Technology Ropar, Rupnagar, Punjab 140001 India; 4 Condensed Matter Theory Group, Department of Physics and Astronomy, Uppsala University, SE-75120 Uppsala, Sweden

**Keywords:** Batteries, Batteries

## Abstract

Transition metal oxides (TMO) are the preferred materials for metal ion battery cathodes because of their high redox potentials and good metal-ion intercalation capacity, which serve as an outstanding replacement for layered sulphide. In this work, using first-principles calculations based on Density functional theory approach, we explored the structural and electronic properties which comprise of adsorption and diffusion behaviour along with the analysis of voltage profile and storage capacity of Ru doped two-dimensional transition metal oxide $MnO_{2}$, $CoO_{2}$, and $NiO_{2}$ monolayers. The adsorption of alkali ions (Li, Na) to the surface of TMOs is strengthened by Ru-atom doping. Ru doping enhanced the adsorption energy of Li/Na-ion by 25%/11% for $MnO_{2}$, 8%/13% for $CoO_{2}$, and 10%/11% $NiO_{2}$ respectively. The open circuit voltage (OCV) also increases due to the high adsorption capacity of doped Monolayers. Ru doping makes the semiconducting TMOs conduct, which is suitable for battery application. As alkali ion moves closer to the dopant site, the adsorption energy increases. When alkali ions are close to the vicinity of doping site, their diffusion barrier decrease and rises as they go further away. Our current findings will be useful in finding ways to improve the storage performance of 2D oxide materials for application in energy harvesting and green energy architecture.

## Introduction

The need for energy resources has grown significantly along with the quick growth of contemporary society. However, burning fossil fuels severely pollutes the environment and depletes non-renewable resources. As a result, it is becoming more and more necessary to find reusable and clean energy sources. Lithium-ion batteries (LIBs) are key components of many energy storage systems and offer a wide range of opportunities for the growth of new energy sectors^1^. The market for lithium-ion batteries (LIBs) as an electric energy source is expanding as a result of the meteoric rise in sales of electric vehicles and portable gadgets. However, the energy density and fast charging-discharging rate have limited their advancements^2^. The energy density of LIBs can be increased by using electrodes made of materials with high kinetics^3^. Therefore, research into novel materials with high-voltage profiles and high energy densities is urgently needed for energy harvesting^4^.

A lot of two-dimensional (2D) materials have similar inherent benefits, for example, the abundant adsorption sites, short metal atom diffusion path, and great mechanical qualities with spin-transport functionality^5,6^ etc. Moreover, large specific surfaces can facilitate ion adsorption and boost capacitance; high conductivity can quicken electron transport; configurable interlayer spacing is advantageous for ion intercalation and customizable active sites can exhibit electrocatalytic activity^7,8^. Due to all of these benefits, 2D materials are potential options for energy storage. 2D transition metal dichalcogenides (TMDs)^9,10^, Janus materials^11^ and transition metal oxides (TMOs)^12^ with characteristic sandwiched structure of few atomic layers^13^ have received a lot of attention recently. Similar to graphene, some TMD materials, such as $MoS_{2}$^14^, $VS_{2}$^15^, $WS_{2}$^16^, $TiS_{2}$^17^, and $SnS_{2}$^18^ that have a single layer or a few layers, have been suggested for usage as electrode materials in LIBs in both tests and theories. The cathode, one of the most crucial parts of metal-ion batteries, affects both the price and capacity of these batteries. Layered bulk metal oxides are currently utilized extensively in commercial LIBs. The creation of well-ordered structures is challenging, and the capacity is decreased by the bulk shapes of these layered materials.

2D oxide monolayers can offer remedies for these problems. Depending on the point-group symmetries, the oxide monolayers may have two different structures, such as 2H and 1T structures for $D_{6}h$ and $D_{3}d$ point-group symmetries, respectively^19^. Theoretically, $MnO_{2}$, $CoO_{2}$ and $NiO_{2}$ with the 1T structure are stable, according to Ataca et al.^20^. Among all the 2D materials, 2D TMOs show a large number of stabilised structures, just because the cations take on many charge states and binding configurations. 2D transition-metal oxide monolayers ($MO_{2}$; M = Mn, Co, and Ni)^12,21^ have already been reported in the energy storage field as a cathode material for Li and Na ion batteries but they are not explored much in the context of rechargeable batteries. After the experimental synthesis of 2D $MnO_{2}$^22^, it opens up many possibilities as an electrode for rechargeable batteries. Similarly, another 2D oxide $RuO_{2}$, although experimentally synthesised but underestimated in the context of electrode application. The reported 1T phase of $RuO_{2}$ monolayer^23^ is half-metallic in nature with a high value of specific capacity with a decent voltage profile. $RuO_{2}$, $SnO_{2}$, and $SnS_{2}$ are already reported in Li-ion batteries applications^18^, among which $RuO_{2}$ monolayer comes with various advantages.

Hetero-atom doping can alter the electrical and chemical properties of the parent structure^24,25^. For example, DFT calculations show a reduction in the ion-diffusion barrier and a narrowing of the bandgap (from insulator to semiconductor), resulting in significantly improved ion/electron conductivities of Mo doped $TiNb_{2}O_{7}$ micro rods^26^. Similarly, a very high capacity of 199 mAh^1^ gm^-1^ and 235 mAh^1^ gm^-1^ was obtained for the N-doped graphene and B-doped graphene^27–29^. After analysing the potential application of Ruthenium oxide ($RuO_{2}$) in the energy storage field in this work for the first time, we have doped Ruthenium(Ru) atom, in transition-metal oxide monolayers ($MO_{2}$; M = Mn, Co, and Ni). In this process of doping, we have substituted transition metal with Ru atom and we are taking the moderate doping percentage that is $\sim$ 6% which can be archived by different chemical processes. We studied the electronic and structural properties where the adsorption of metal atoms at different active sites was systematically studied, along with the diffusion behaviour, specific capacity, and open circuit voltage was calculated by adopting the first principles based density functional theory approach.

## Computational methods

First-principles based calculations were performed using Quantum ATK^30^ which uses numerical linear combinations of atomic orbitals (LCAO) basis sets as implemented within Density functional theory approach. We have adopted the spin-polarized generalized gradient approximation (SGGA) using the Perdew-Burke-Ernzerhof parametrization technique (PBE technique) to take the electron exchange-correlation interactions^31^ into consideration. In order to investigate the potential consequences of on-site Coulomb interaction of localized electrons in the investigated transition metals, we used DFT+U calculations^32,33^. A density mess cut-off of 125 in the Hartree unit was employed for the plane wave basis sets. A Monkhorst-Pack *k*-grid with a 5 $\times$ 5 $\times$ 1 k-point mesh was adopted for geometry optimization. A 22 Å  vacuum zone was applied in the out-of-plane direction to prevent interaction between periodic images. For structural relaxations, the energy and maximum force convergence criteria were set at $10^{-5}$ per atom and 0.01 eV Å^-1^, respectively. In the process of calculating the band structure and density of states, the medium basic set with PseudoDojo pseudopotential was used, and a Monkhorst-Pack grid of 7 $\times$ 7 $\times$ 1 points was used to build the Brillouin zone. The thermal stability of the pristine as well as metal atom adsorbed systems was addressed by performing Ab initio molecular dynamics (AIMD) calculations. To identify the charge transfer between the Metal-ion and TMOs monolayers, Bader charge analysis and isosurface charge density difference plot were performed with a densed 25 $\times$ 25 $\times$ 25 FFT grid, where we calculated the charge transfer by plotting the isosurface plot. The climbing-image nudged elastic band (CI-NEB) approaches were utilized to identify the shortest energy pathway between the specified initial and final configurations in order to study the metal atom diffusion behaviour on the monolayer.

## Results and discussion

### Lattice structure and electronic properties of pristine transition-metal oxide monolayers ($MO_{2}$, M = Mn, Co, and Ni)

**Figure 1:**
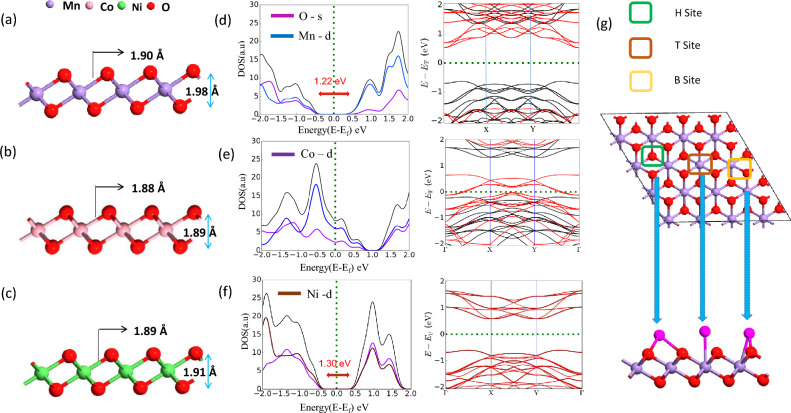
(**a**), (**b**) and (**c**) are the side view of pristine $MnO_{2}$, $CoO_{2}$, and $NiO_{2}$, (**d**), (**e**), (**f**) band structure and PDOS of $MnO_{2}$, $CoO_{2}$, and $NiO_{2}$ respectively, (**g**) possible adsorption sites for metal atom.

The monolayer has a D3d point-group symmetry and is in a sequence of an O-M-O layer that is three atoms thick where the Mn layer is sandwiched between two O layers^34^. The $MnO_{2}$, $CoO_{2}$, and $NiO_{2}$ monolayers can be grown by the molecular beam epitaxy method and these structures are comparable to transition metal dichalcogenides (TMD) in the T phase^35^, where each transition metal atom occupies the centre of an octahedron made of six O atoms (Fig. 1a–c,g). The monolayers $MnO_{2}$^36^ and $NiO_{2}$ are ferromagnetic semiconductors and we have calculated the band gap of 1.22 eV and 1.30 eV respectively. On the other hand, $CoO_{2}$ monolayer is magnetic and half-metallic in nature^37^. We took a 4 $\times$ 4 $\times$ 1 supercell of monolayers

to design our structure which contains 48 atoms to save computational time. The projected density of states (PDOS) of $MnO_{2}$, $CoO_{2}$, and $NiO_{2}$ are shown in Fig. 1(d–f). The bond length between Manganese and Oxygen was taken to be 1.90 Å . The bond length between Co and oxygen and Ni and oxygen is set to be 1.88 Å . and 1.89 Å Fig. 1(a–c). The lattice parameter of $MnO_{2}$ is 11.26 Å and $CoO_{2}$ and $NiO_{2}$ are 11.29 Å respectively.

We have calculated the adsorption behaviour of pristine TMO monolayers by putting the alkali metal atom (Li and Na) on the monolayer’s surface. For the adsorption behaviour, we took three favourable adsorption sites as shown in Fig. 1g. Among the adsorption sites, the B site is the metastable site, i.e. after optimization metal atom present on the B site comes to the T site whereas the metal atom present on H and T sites are representing stable sites showing no displacement of the Li and Na atom after optimization. We have calculated the PDOS of single metal atom adsorbed on pristine TMO monolayers which are shown in Fig. S2^38^ in the supporting information.

### Structural, electronic properties and stability of the Ru doped 2D $MnO_{2}$, $CoO_{2}$, and $NiO_{2}$ monolayers

**Figure 2:**
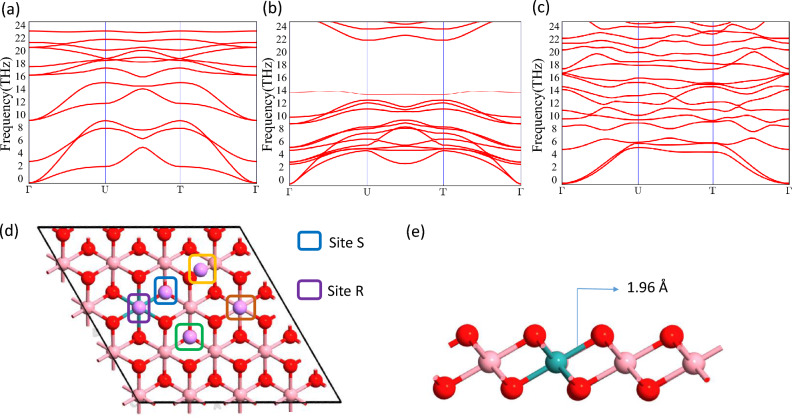
(**a**), (**b**), (**c**) Phonon Band structure of Ru doped 2D $MnO_{2}$, $CoO_{2}$, and $NiO_{2}$, (**d**) Horizontal view with possible adsorption sites, (**e**) Side view of doped monolayer.

Before the analysis of electronic properties, the structural stabilities of all the monolayers should be explored after the substitution of transition metal atoms with Ru atoms. It was reported that it is possible to increase the catalytic activity of a host metal oxide by substituting a small portion of the cations with another cation also the transition metal (Fe, Co, Ni, and Cu)-doped $\alpha$-$MnO_{2}$ nanowires^39^ synthesized by a one-step hydrothermal method. So it opens up possibilities of doping in TMOs for different applications. We took into account a doping level of 6%, which is equivalent to doping one metal atom on a 4 $\times$ 4 $\times$ 1 supercell (with 16 Transition metal atoms and 32 O atoms). For reference we have shown the horizontal view of the Ru doped $CoO_{2}$ monolayer in Fig. 2(e). One indicator of a structure’s stability is formation energy which is calculated using the following equation^40^.

1 $$\begin{aligned} E_{form} = { E_{doped} - E_{pristine} + E_{M}-E_{Ru}} \end{aligned}$$

where $E_{doped}$ is the total energy of the doped TMOs and $E_{pristine}$ is the total energy of undoped monolayers, $E_{M}$ and $E_{Ru}$ are the total energy of transition metal and Ru atom in its pure form respectively. The calculated formation energy for all the doped TMOs monolayers is negative indicating the stabilities of respective monolayers. The formation energy of all the monolayers is mentioned in Table 1. We also analysed the adsorption of the Ru atom on pristine monolayers and we conclude that the adsorption energy is more than the formation energy indicating doping is more suitable than the adsorption of the Ru atom. To explore the possibilities of stability of our structure, we have calculated the Phonon band structure shown in Fig. 2(a–c). As no negative frequency appears in the phonon dispersion in all the doped structures, they are found to be stable. However the thermal stability of the transition metal oxide (TMOs) monolayers is a major concern, so we have performed Ab initio molecular dynamics calculation at room tempreature to predict the thermal stabilities of the Ru doped TMOs monolayers Fig. (S6)^38^. From the AIMD calculations we can see the energy fluctuations are very small which indicate the stability at ambient tempreature. From the final structure of Ru doped $MnO_{2}$, We can see some extra bond present between Ru and Mn atom which were not present in the pristine monolayer. However for Ru doped $CoO_{2}$ and $NiO_{2}$ monolayer no such bonds were seen. Also, the bond length between Ru atom and the O atom appears to be more than the bond length of the previous Transition metal and O. Ru doping displays metallic properties, Ru atoms serve as electron donors and supply free electrons, resulting in higher electronic states in each case. We have also briefly studied the effect of Mo, Nb, and W doping in TMOs for analyzing the structural and electronic properties. The optimized Mo, Nb, and W-doped TMO structures are shown in Fig. S1^38^. of the supporting information.

### Adsorption behavior of alkali metal atom

We first identify the most favorable position for a single metal atom adsorption on the monolayers in order to explore the nature of the adsorption of an alkali or alkaline earth metal M (= Li, Na) atom. From the symmetry of the structure, we have considered two new adsorption sites (S site, R site) along with H, T, and B sites Fig. 2d. The metal atom present in H and R sites is bonded with three oxygen atoms i.e. hollow site (on an O atom in the bottom layer) whereas the metal atom present on S and T sites, one single bond can be seen between the metal atom and transition metal or doped atom. The adsorption energy was calculated with the following formula^41^

2 $$\begin{aligned} E_{ad} = \frac{{E_{doped+M} - E_{doped}-nE_{M}}}{n} \end{aligned}$$

where $E_{doped+M}$ is the total energy of the doped TMOs with a single atom and $E_{pristine}$ is the total energy of undoped monolayers, $E_{M}$ is the energy of the isolated metal atom, n is the number metal atoms. A more negative adsorption energy indicates a more favourable exothermic reaction between monolayer and metal atoms^42^.

**Table 1:** adsorption distance of Li ($d_{Li}$) Åand Na ($d_{Na}$) atoms, on doped TMOs monolayer, Formation energies ($E_{form}$), adsorption energies $E_{ad}$(Li/Na), and the enhancement in adsorption $\epsilon$ (Li/Na) from pristine and dQ is the charge transfer in unit of e.

TMO	$d_{Li}$(Å)	$d_{Na}$(Å)	$E_{form}$	$E_{ad}$ (eV)	$\epsilon$ (%)	dQ(e)
			(eV)	Li/Na	Li/Na	Li/Na
$MnO_{2}$	1.53	1.88	2.89	− 3.07/-2.14	25/11	0.31/0.32
$CoO_{2}$	1.50	1.81	1.75	− 3.64/-2.58	8/13	0.44/0 38
$NiO_{2}$	1.59	1.93	1.37	− 3.16/-2.13	10/11	0.38/0.31

**Table 2:** Adsorption energy (eV) of lithium on different adsorption sites respectively.

Ru doped TMO	$E_{ad}$ (R)	$E_{ad}$ (H)	$E_{ad}$ (S)	$E_{ad}$ (T)
$MnO_{2}$	− 3.07	− 2.97	− 2.89	− 2.75
$CoO_{2}$	− 3.64	− 3.53	− 3.37	− 3.28
$NiO_{2}$	− 3.16	− 3.07	− 2.90	− 2.11

**Table 3:** Adsorption energy (eV) of sodium on different adsorption sites respectively.

Ru doped TMO	$E_{ad}$ (R)	$E_{ad}$ (H)	$E_{ad}$ (S)	$E_{ad}$ (T)
$MnO_{2}$	− 2.14	− 2.10	− 2.07	− 2.00
$CoO_{2}$	− 2.58	− 2.44	− 2.33	− 2.20
$NiO_{2}$	− 2.13	− 2.07	− 1.97	− 1.85

Site R is the energetically preferred adsorption site for all metal atoms due to the highest negative adsorption energy value whereas site H has the next higher energy followed by T site, indicating adsorption energy goes down when we leave the vicinity of the doped Ru atom. The R site can be attributed to the more number of M-O bonds (Three Metal atom-O bonds). Metal atoms will strongly adhere to the monolayer because of the extremely negative adsorption energies.

From Table 1 it was clear that Ru-doped TMO monolayers show enhancement in the adsorption of metal atoms. The adsorption energy of Ru doped $MnO_{2}$ increased from  − 2.29 eV of the pristine monolayer to  − 3.07 eV, similarly for doped $CoO_{2}$ and $NiO_{2}$ adsorption energy increased from  − 3.36 eV to  − 3.64 eV and  − 2.82 eV to  − 3.16 eV for Li and  − 1.89 eV to  − 2.14,  − 2.24 eV to 2.58 and  − 1.90 eV to  − 2.13 eV for Na atom respectively. These adsorption values are much greater than some well known electrode materials (FeSe^43^, $NbSe_{2}$^44^, phosphorene^45^, borophene^46^ and Janus monolayers^47,48^). Also, the increment of the percentage of adsorption energy is noted in Table 1 which was calculated using the following formula,

3 $$\begin{aligned} \epsilon = \frac{{E(ad)_{doped} - E(ad)_{pristine}}}{E(ad)_{doped}} \end{aligned}$$

where $E(ad)_{doped}$ and $E(ad)_{pristine}$ are the adsorption energy on doped and pristine monolayers for metal atoms.

As concluded, for Ru-doped $MnO_{2}$ adsorption energy for Li atom is significantly increased (25%) indicating better intercalation. The adsorption distances of the alkali atoms from the monolayers are summarized in Table 1 where the adsorption distances $d_{Li}$ and $d_{Na}$were measured from the top layer formed by O atoms. The Na atom adsorbed at a larger distance as compared to the Li atom for a particular monolayer. This can be confirmed from the charge transfer of Li and Na. As Na atom transfers less charge to the monolayer, we expect Na interacts weakly with the Ru doped monolayer as compared to Li. One of the expected reason is also that Na atom is larger in size as compared to Li atom, so Na needs larger space to accomodate in between the layers formed by Ru doped TMO monolayers. The adsorption energy of lithium and sodium on different adsorption sites is mentioned in Tables 2 and  3 respectively. We compared the adsorption energy of Li and Na for Nb, W, and Mo-doped TMOs which are mentioned in Fig. S4^38^ in the SI. Comparing all the doping agents it was concluded that Ru doped TMOs show more enhancement of adsorption energy compared to others.

**Figure 3:**
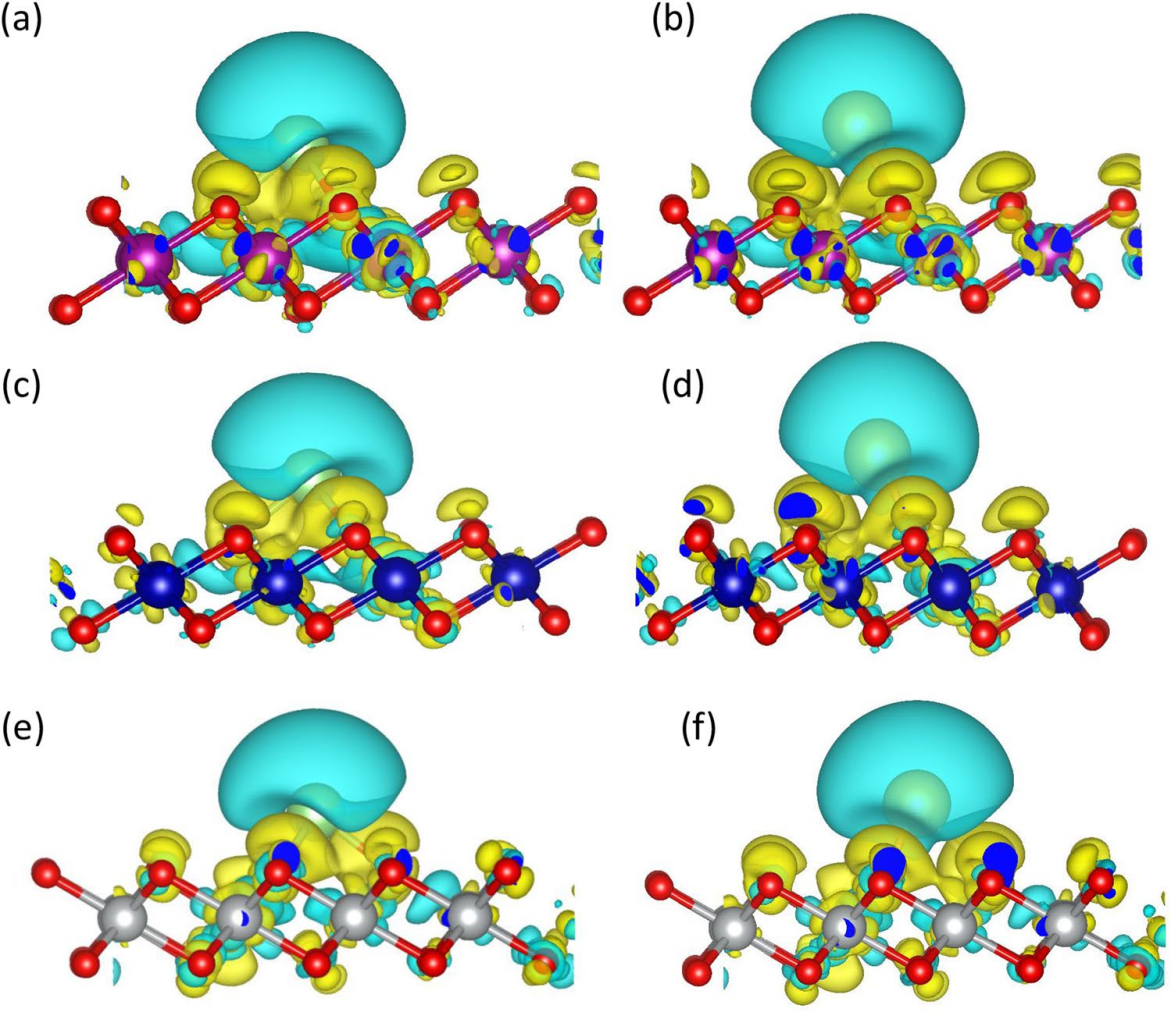
(**a**), (**b**) iso-surface plot for Li and Na adsorbed Ru doped $MnO_{2}$ (**c**), (**d**) and (**e**), (**f**) iso-surface plot for Li and Na adsorbed Ru doped $CoO_{2}$ and $NiO_{2}$ monolayers respectively. Yellow and cyan colors represent electron accumulation and electron depletion regions respectively.

**Figure 4:**
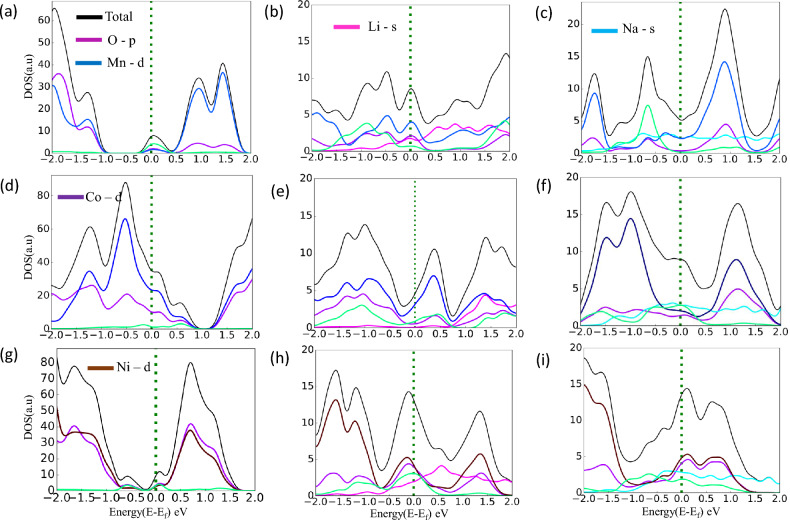
(**a**), (**b**), (**c**) PDOS of Ru doped $MnO_{2}$ without intercalation and Full Lithium and Sodium intercalated structure (**d**), (**e**), (**f**) and (**g**), (**h**) and (**i**) represent PDOS of Ru doped $CoO_{2}$ and $NiO_{2}$ monolayer without and full intercalation of Li and Na atom respectively.

The isosurface plot of Li and Na adsorption on the Ru-doped monolayer surfaces is shown in Fig. 3 to visualize the adsorption process in more details. The charge transfer of Li/Na atom to Ru doped $MnO_{2}$, $CoO_{2}$ and $NiO_{2}$ on the R site are 0.31e/0.32e, 0.44e/0.38e and 0.38e/0.31e respectively which are confirmed from Brader charge analysis. According to the charge transfer of around 0.3e to 0.45e, there was a considerable electron transfer from the metal atom to the nearby O atoms, indicating a stronger bond between the metal atom and the TMO surface. The PDOS of single metal atom adsorbed in Ru-doped TMOs are shown the Fig. S3^38^ in the SI. As the specific capacity and open circuit voltage are the outcome of full adsorption, we fill the structure with metal atoms on the R site first, which is the most favourable site, then on H Site, and optimized the structure for further calculation. To visualize the contribution of different orbitals to the density of states we have calculated the band structure and projected density of states for all the doped structures before and after the intercalation of metal atoms as shown in Fig. 4.

From PDOS it can be cleared that the d orbital of Mn, Co, Ni, and Ru whereas the s orbital of Li and Na are contributed significantly to the band structure. The PDOS of Mo, Nb, and W-doped TMO structures are given in Fig. S5^38^ in the SI.

### Diffusion of single metal atoms (Li and Na)

**Figure 5:**
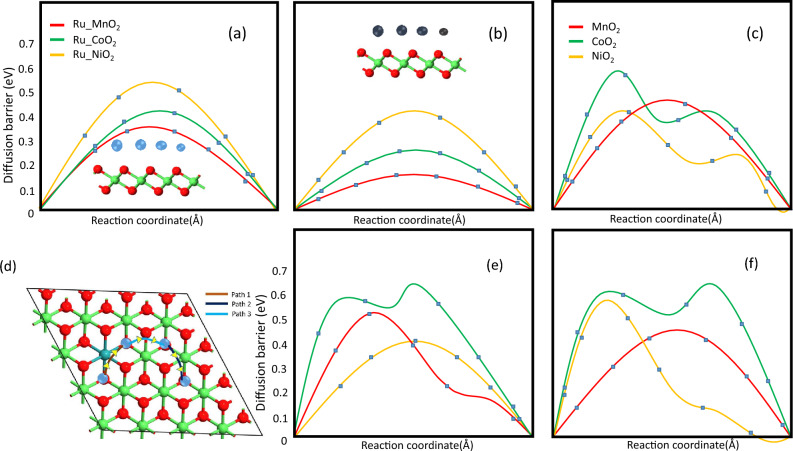
(**a**), (**b**) represent Diffusion barrier on Ru doped TMOs monolayers along path 1 for Li and Na respectively, (**c**) lowest diffusion barrier of pristine TMOs, (**d**) visualization of diffusion path (**e**), (**f**) diffusion barrier of Li and Na through path 2.

An essential metric for assessing electrode materials is the charge-discharge rate, which is mostly determined by the diffusion barrier of metal atoms^49^. Using the CI-NEB approach, we investigated the metal ion transport on the Ru-doped transition metal oxide monolayers. We took into consideration three different diffusion channels by analysing the symmetry and adsorption energy of TMO monolayers. Path 1 refers to the diffusion of metal ion from the R site to another R site via the B site of Ru and transition metal atom whereas path 2 and path 3 show the diffusion from H site to the R site and the H site to another H site via the B site respectively. B site as a metastable site serves as a channel for the diffusion process. We have calculated relative diffusion mobility using the Arrhenius equation. The diffusion constant (D) of Lithium and sodium ion can be calculated using the following equation^50^.

4 $$\begin{aligned} D = D_{0}e^{\frac{-E_{b}}{K_{B}T} } \end{aligned}$$

where T is the ambient temperature, $E_{b}$ is the barrier energy, $k_{B}$ is Boltzmann’s constant, and $D_{o}$ is the temperature-independent pre-exponential constant and we took the $D_{o}$ value same for all paths.

The calculated diffusion barrier for Path 1 is found to be the lowest among all other paths, representing metal atoms prefer to travel around the neighbourhood of Ru atom, the next preferable path is Path 2 followed by Path 3. The lowest diffusion barrier for the pristine $MnO_{2}$, $CoO_{2}$, and $NiO_{2}$ are found to be 0.5 eV, 0.6 eV, and 0.4 eV respectively whereas, for the Ru-doped TMOs, the diffusion barrier is found to be 0.35 eV, 0.42 eV, 0.52 eV for Li-ion and 0.11 eV, 0.28 eV and 0.42 eV for Na atom respectively which indicates that doping lowers the barrier to promote fascinating ion diffusion.

**Table 4:** Calculated diffusion barrier($E_{b}$) eV for 3 different paths for Li and Na.

Ru doped TMO	$E_{b}$ (Path 1)	$E_{b}$ (Path 2)	$E_{b}$ (Path 3)
	(Li/Na)	(Li/Na)	(Li/Na)
Ru-$MnO_{2}$	0.35/0.10	0.50/0.30	0.40/0.22
Ru-$CoO_{2}$	0.40/0.20	0.70/0.30	0.70/0.30
Ru-$NiO_{2}$	0.50/0.40	0.40/0.30	0.58/0.30

The calculated diffusion barrier for all the paths for Li and Na are mentioned in Table 4. The diffusion barrier for sodium is found to be less than the diffusion barrier for lithium. This was expected for the larger adsorption distance of Na as compared to lithium. The diffusion barrier of alkali metal atoms on different paths is shown in Fig. 5. The calculated diffusion barrier for our system is lower than the some of the well reported structure $Mg_{3}N_{2}$^51^, $B_{3}S$^52^.These small barrier allow the fast movement of the metal atom which in turn fasten the charging capability.

### Open circuit voltage and specific capacity

It is necessary for the cathode materials to work in conjunction with electrodes in applications because they are one of the key elements of lithium-ion batteries. The positive electrode (Cathode) must have a comparatively high open-circuit voltage in order to create a high electric potential^53,54^. To ensure higher output voltage, the positive electrode material frequently needs to: (i) have high and stable potential, (ii) maintain its structural stability after full adsorption of metal atoms during charging, (iii) have higher specific capacity. By analysing the properties while gradually increasing metal atoms, these two properties are evaluated. We used the 4 $\times$ 4 $\times$ 1 supercell of the monolayers as the substrate.

**Table 5:** Comparision of O.C.V (Volt) between pristine and doped structure.

Pristine	O.C.V	Ru doped	O.C.V
TMO	(Li/Na)	TMO	(Li/Na)
$\hbox {MnO}_{{2}}$	1.87/1.1	MnO_2_	2.02/1.23
CoO_2_	2.10/1.31	CoO_2_	2.30/1.35
NiO_2_	2.30/1.34	NiO_2_	2.43/1.43

which allowed us to make changes in the charging process to the adsorption of metals on both sides of the monolayers until it achieves its full capacity. The interaction between the adsorbed layers caused the absolute value of the average adsorption energy to steadily decrease as the amount of Li increased. The change of open circuit voltage with the metal atom content is shown in Fig. 6. The OCVs of Li and Na atoms are calculated according to the following equation by using average adsorption energy, $E_{ad}$ of n metal atoms adsorbed system, and e is the charge of an electron.

5 $$\begin{aligned} OCV = -\frac{E_{ad}}{e} \end{aligned}$$

where $E_{ad}$ is the average adsorption energy of a metal atom and e is the charge of an electron.

The calculated O.C.V for all three Ru-doped monolayers is mentioned in Table 5. It was clear that Ru-doped $MnO_{2}$, $CoO_{2}$, and $NiO_{2}$ showed enhancement in cell voltage as compared to pristine monolayers, which is a desirable feature for the next-generation cathode material. However some article have followed different apporach to calculate the O.C.V which we have discussed in the last section of the supplimentary information in Fig. S9^38^. The specific capacity for Lithium and Sodium storage can be estimated using the following equation,

6 $$\begin{aligned} C = \frac{xnF}{M + nM_{m}} \end{aligned}$$

Here M is the molecular weight of doped monolayers, $M_{m}$ is the molecular mass of the metal atom and F is the Faraday constant of 26801 mAhmol^-1^, x is the chemical stoichiometry of the metal atom and n is the valency of lithium and sodium ion i.e n=1 for Lithium and Sodium, n=2 for potassium, Calcium.

**Figure 6:**
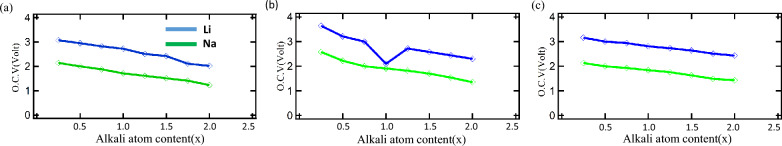
(**a**), (**b**), (**c**) Open circuit voltage Vs content of Li and Na atom in Ru doped $MnO_{2}$, $CoO_{2}$ and $NiO_{2}$respectively.

The increased Li concentration provides more repulsive interaction, therefore the Li adsorption energy steadily declines as x increases. The metal atom concentration in the Ru-doped TMO monolayers reaches the maximum value, which corresponds to the case of x = 2 in $Li/Na_{x}MO_{2}$. At this stage the calculated specific capacity for Ru doped $MnO_{2}$, $CoO_{2}$, and $NiO_{2}$ are 453 mAhgm^-1^/361 mAhgm^-1^, 442 mAhgm^-1^/357 mAhgm^-1^ and 441 mAhgm^-1^/355 mAhgm^-1^for Li/Na. Thermal stability of the electrode material after full lithiation and sodiation is most important. To check the thermal stability at room temperature we have performed Ab initio molecular dynamics (AIMD) calculations. The AIMD calculation results for full lithium and sodium intercalated system for all three Ru-doped 2D $MnO_{2}$, $CoO_{2}$, and $NiO_{2}$ monolayers are shown in Figs. S7, S8^38^. We observed a very little energy fluctuation, so the structures are expected to be stable.

## Conclusion

We have thoroughly analyzed the performance of pristine and Ru-doped 2D $MnO_{2}$, $CoO_{2}$, and $NiO_{2}$ monolayers as cathode material for lithium- and sodium-ion batteries using DFT calculation. Our findings reveal that Ru doping enhanced the adsorption energy of the monolayers. $MnO_{2}$ showed the highest 25% increment in adsorption while $CoO_{2}$ and $NiO_{2}$ showed an enhancement of 13% and 11% respectively which represent strong binding of alkali ions as compared to pristine monolayers. The adsorption energy increases when one approaches towards the doping site and decreases when one moved away from the doping site showing doping sites are more favourable for metal atom binding. We have also calculated the diffusion barrier along different paths among which metal atoms prefer the path through the vicinity of the doped agent referring to doping facilitating the diffusion process. The lowest barrier for doped $MnO_{2}$, $CoO_{2}$, and $NiO_{2}$ are 0.35 eV/0.14 eV, 0.5 eV/0.3 eV and 0.4 eV/0.22 eV for Li/Na atom respectively. Our findings showed that Ru atom doping can significantly enhance the adsorption and diffusion properties of Transition metal oxide monolayers, making them desirable materials for cathodes in alkali-ion rechargeable batteries.

## Supporting information

The Supporting Information is available here, which contains additional information/data related to teh claims made in the main article^38^.

### Supplementary Information

Supplementary Information.

## Data Availability

The data that support the findings of this study are available from the corresponding author(s) upon reasonable request.
