# Supplementary material for: Remarkable enhancement of the adsorption and diffusion performance of alkali ions in two-dimensional (2D) transition metal oxide monolayers via Ru-doping
Source: Sci Rep. 2024 Feb 22;14:4371. doi: 10.1038/s41598-024-53966-5 (PMC10883979; doi:10.1038/s41598-024-53966-5)
Supplement: Supplementary file 1 — Supplementary Information. [file 41598_2024_53966_MOESM1_ESM.pdf]

# Supplementary Information

## Remarkable Enhancement of the adsorption and diffusion performance of alkali ions in Two-dimensional (2D) Transition metal oxide monolayers via Ru-doping

Shubham Sahoo[1], P. Kumari[1], Narayan N Som[2], S. Kar[1], R. Ahuja[3,4], Soumya Jyoti Ray*[1]

[1]Department of Physics, Indian Institute of Technology Patna, Bihta, Bihar, 801106

[2]Laboratory Nanostructures Institute of High Pressure Physics, Polish Academy of Sciences Sokolowska, Warsaw, Poland

[3]Department of Physics, Indian Institute of Technology Ropar, Rupnagar, Punjab 140001, India

[4]Condensed Matter Theory Group, Department of Physics and Astronomy, Uppsala University, SE-75120, Sweden,

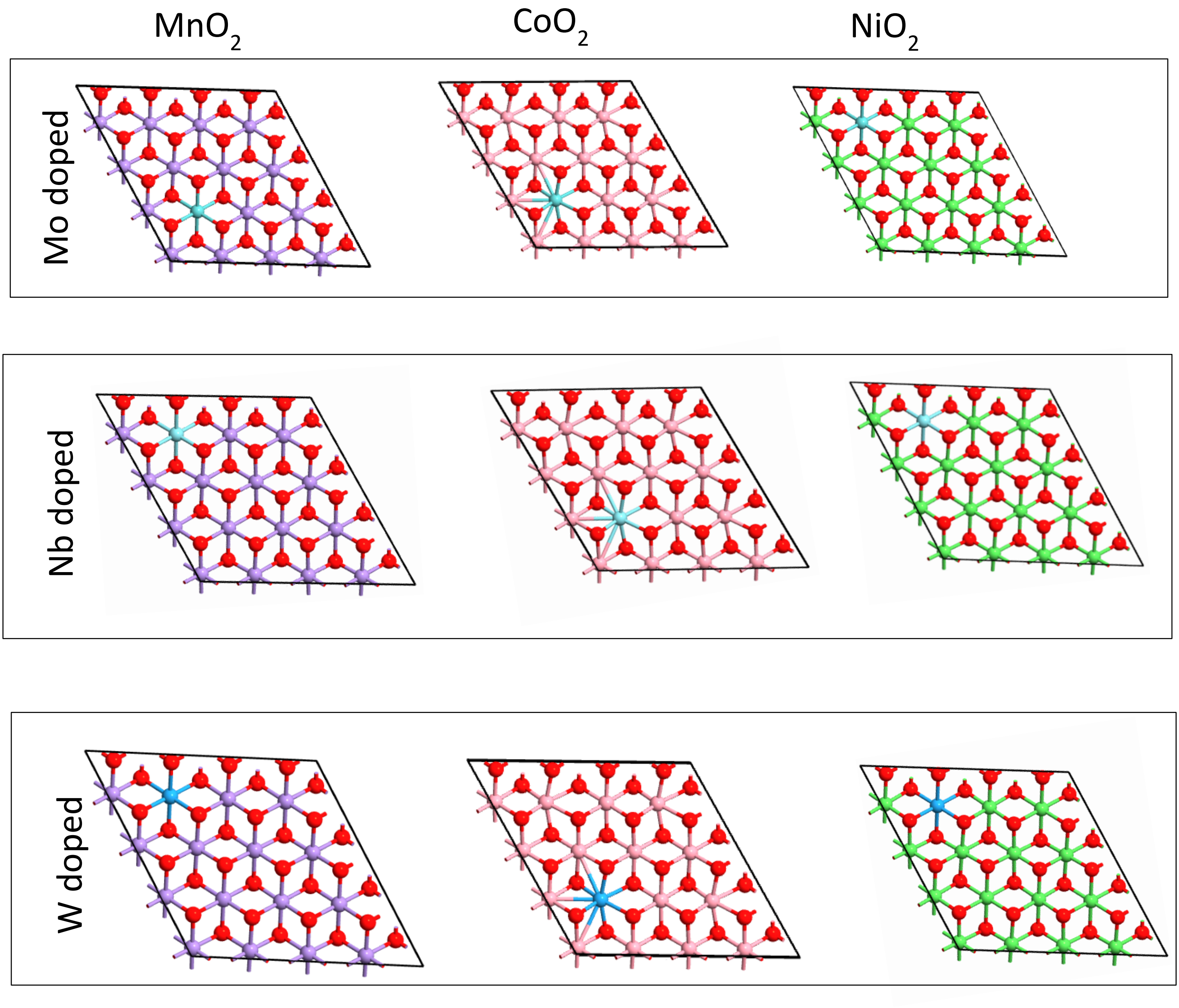


**Fig S1:** Mo, Nb and W doped $MnO_2$, $CoO_2$ and $NiO_2$ monolayer

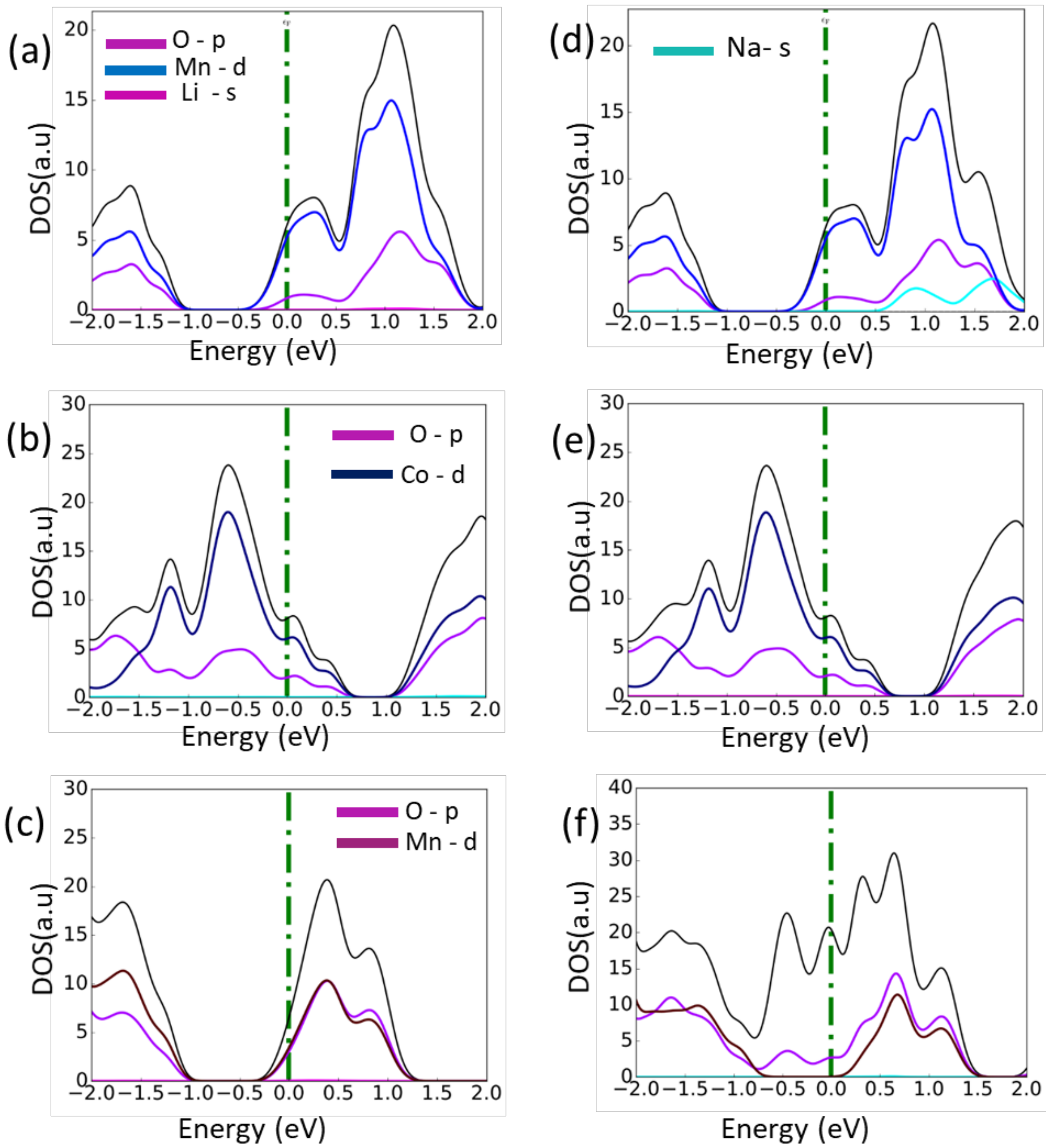


**Fig S2:** (a), (b) and (c) PDOS of single Li adsorbed and (d), (e) and (f) PDOS of single Na adsorbed on Ru doped $MnO_2$, $CoO_2$, and $NiO_2$ monolayers respectively with fermi level set at 0 eV.

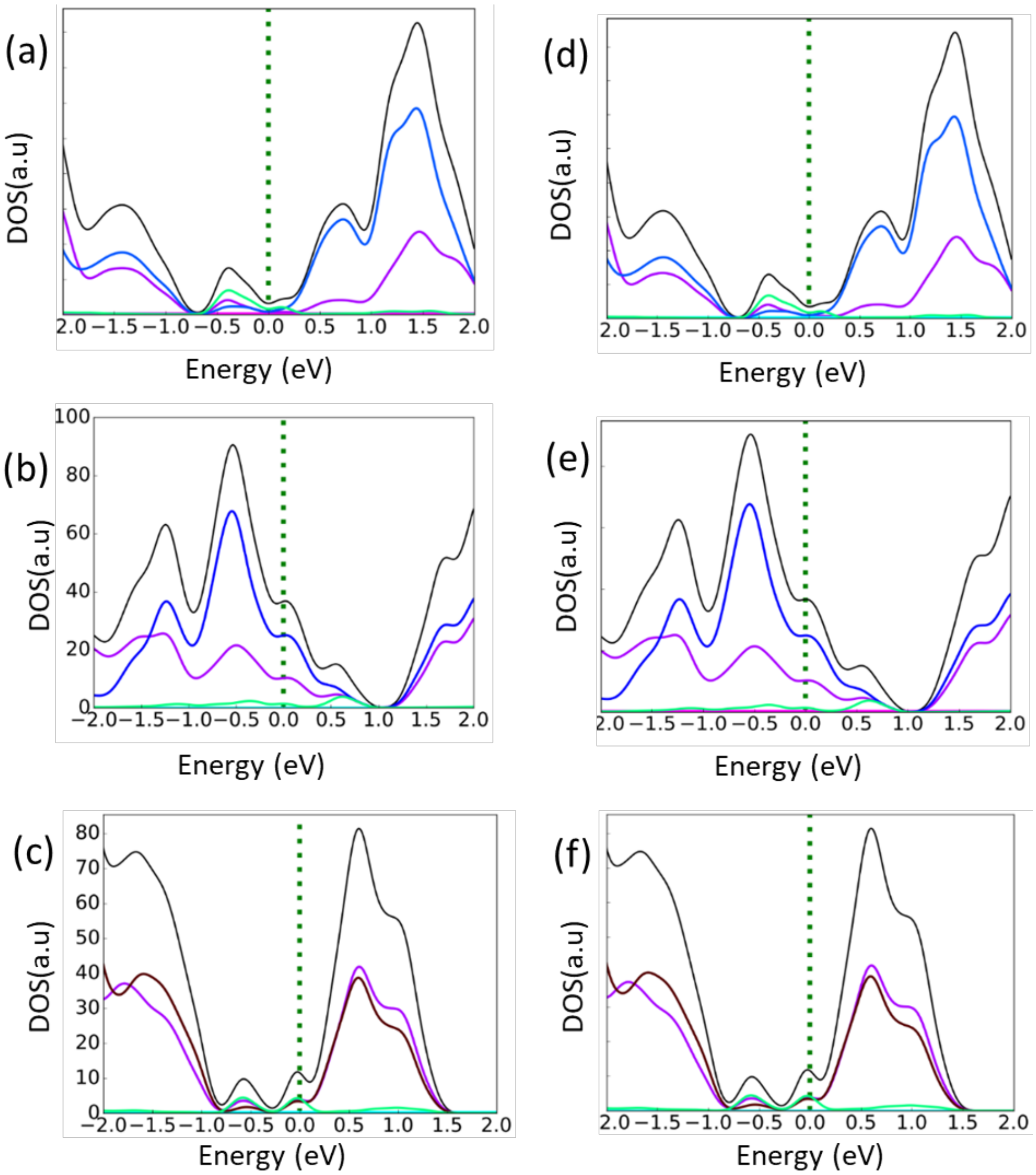


**Figure S3**: (a), (b) PDOS of Li and Na adsorption on Ru-doped $MnO_2$, (c),(d) and (e),(f) PDOS of Li and Na adsorption on Ru-doped $CoO_2$ and $NiO_2$ respectively with fermi level set at 0 eV.

| Mo doped TMO | Adsorption energy in eV | |
|---|---|---|
| | Li | Na |
| $MnO_2$ | -2.54 | -2.00 |
| $CoO_2$ | -3.31 | -2.21 |
| $NiO_2$ | -2.92 | -2.04 |

(a)

| Nb doped TMO | Adsorption energy in eV | |
|---|---|---|
| | Li | Na |
| $MnO_2$ | -2.27 | -2.11 |
| $CoO_2$ | -3.05 | -2.41 |
| $NiO_2$ | -2.91 | -1.99 |

(b)

| W doped TMO | Adsorption energy in eV | |
|---|---|---|
| | Li | Na |
| $MnO_2$ | -2.36 | -2.21 |
| $CoO_2$ | -3.26 | -2.50 |
| $NiO_2$ | -2.96 | -2.09 |

(c)

**Figure S4** : (a), (b), and (c) adsorption energy of Li and Na on Mo, Nb, and W doped TMOs respectively

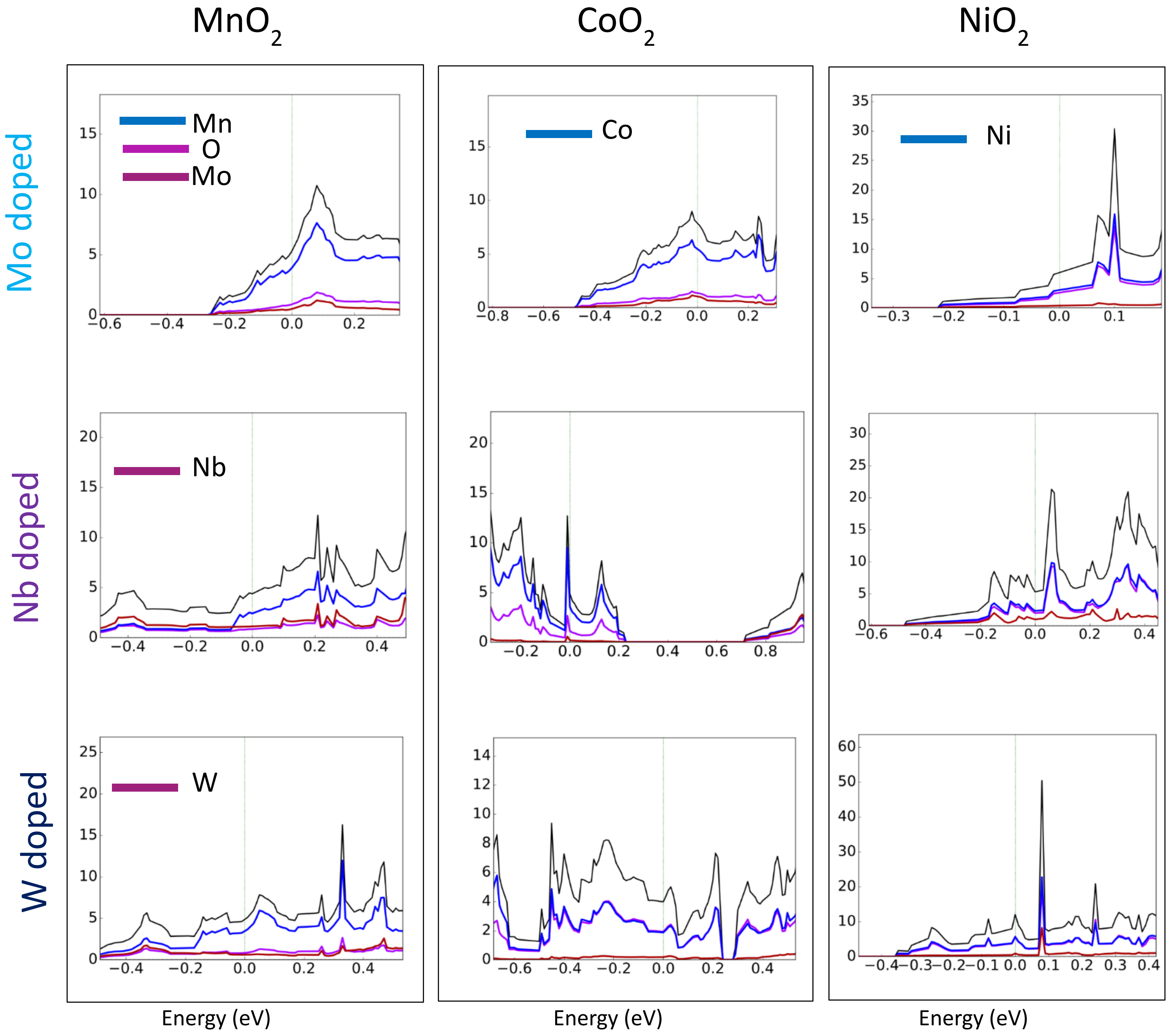

**Figure S5:** PDOS of Mo, Nb and W doped $MnO_2$, $CoO_2$ and $NiO_2$ monolayers respectively with fermi level set at 0 eV .

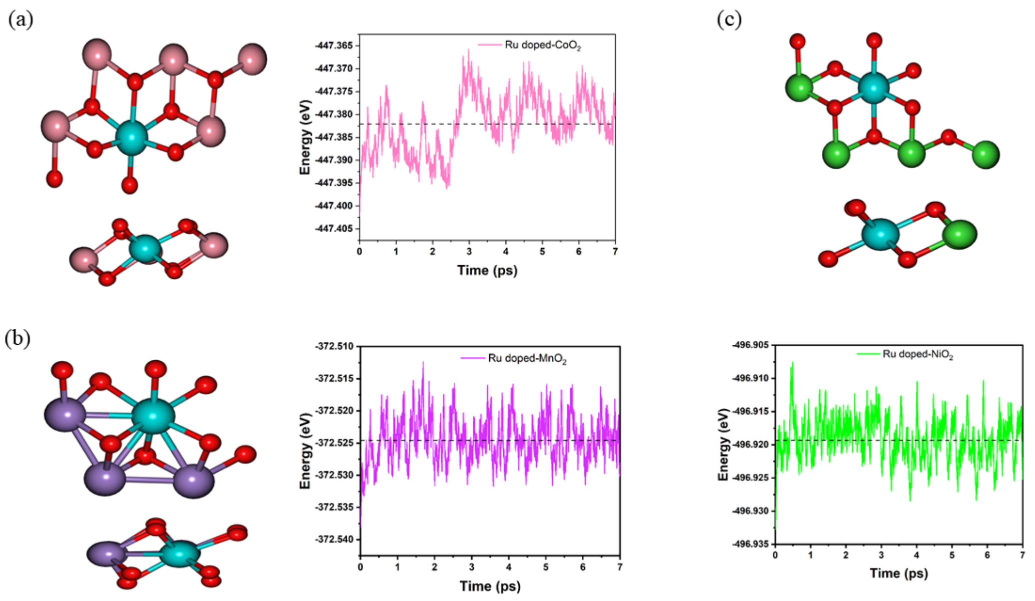


**Fig S6:** (a), (b) and (c) Energy vs Time fluctuations during AIMD simulation for Ru-doped $MnO_2$, $CoO_2$, and $NiO_2$ monolayers with the final structures at room temperature

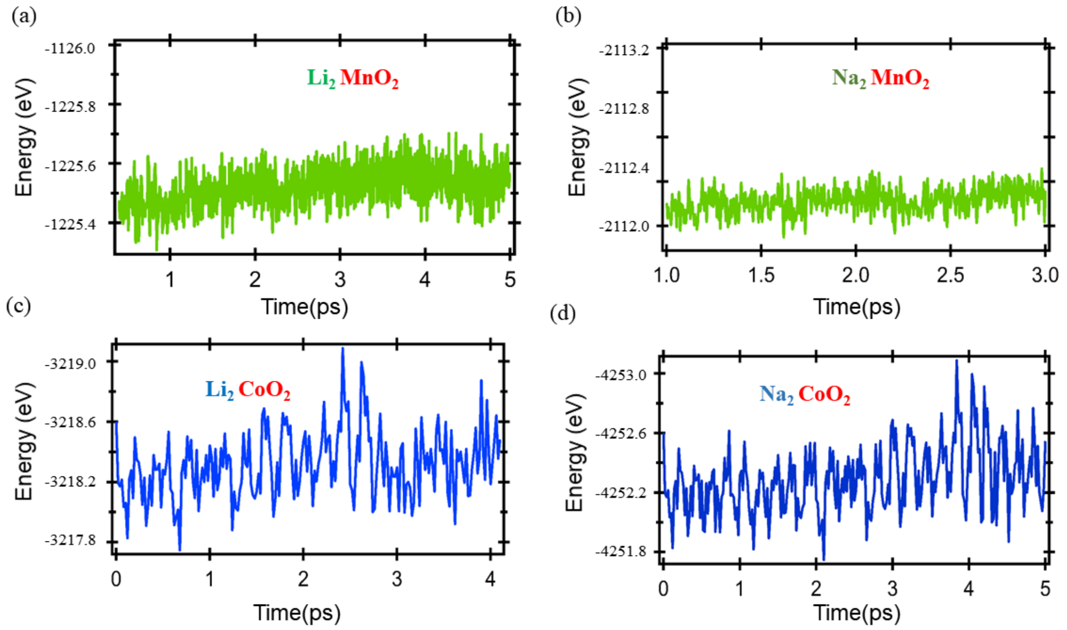


**Fig S7:** (a) and (b) Energy vs Time fluctuations during AIMD simulation for full adsorbed Li and Na in Ru doped $MnO_2$, (c) and (d) AIMD curve for full adsorbed Li and Na in Ru doped $CoO_2$

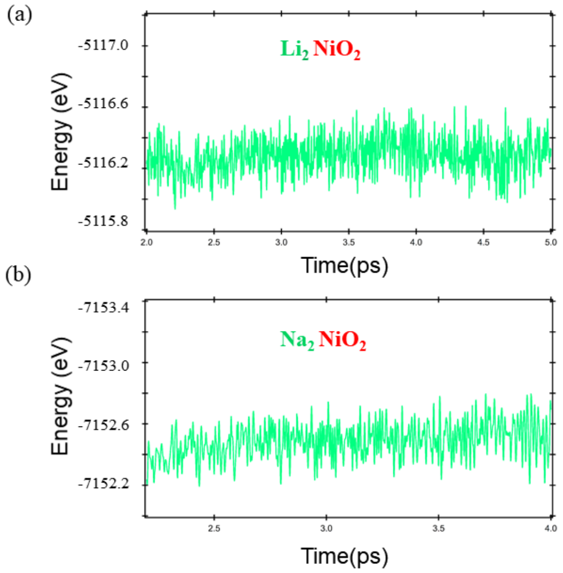


**Fig S8:** (a), (b) Energy vs Time fluctuations during AIMD simulation for full adsorbed Li and Na in Ru-doped $NiO_2$

## <u>O.C.V Vs Li/Na contents</u>

$$\mathrm{OCV} = -\frac{E_{Li/Nax_2Ru-TMO} - E_{Li/Nax_1Ru-TMO} - (x_2-x_1)\,E_{Li/Na}}{(x_2-x_1)}$$

We have used the above formula for the calculation of O.C.V.

Where,

$E_{Li/Nax_2Ru-TMO}$ and $E_{Li/Nax_1Ru-TMO}$ are total energy of $x_2$ and $x_1$ stoichiometry of Li/Na concentration in Ru doped TMO monolayers, $E_{li/Na}$ is the total energy of Li/Na atom in their body center cubic phase. The O.C.V vs metal atom content is shown in the figure below.

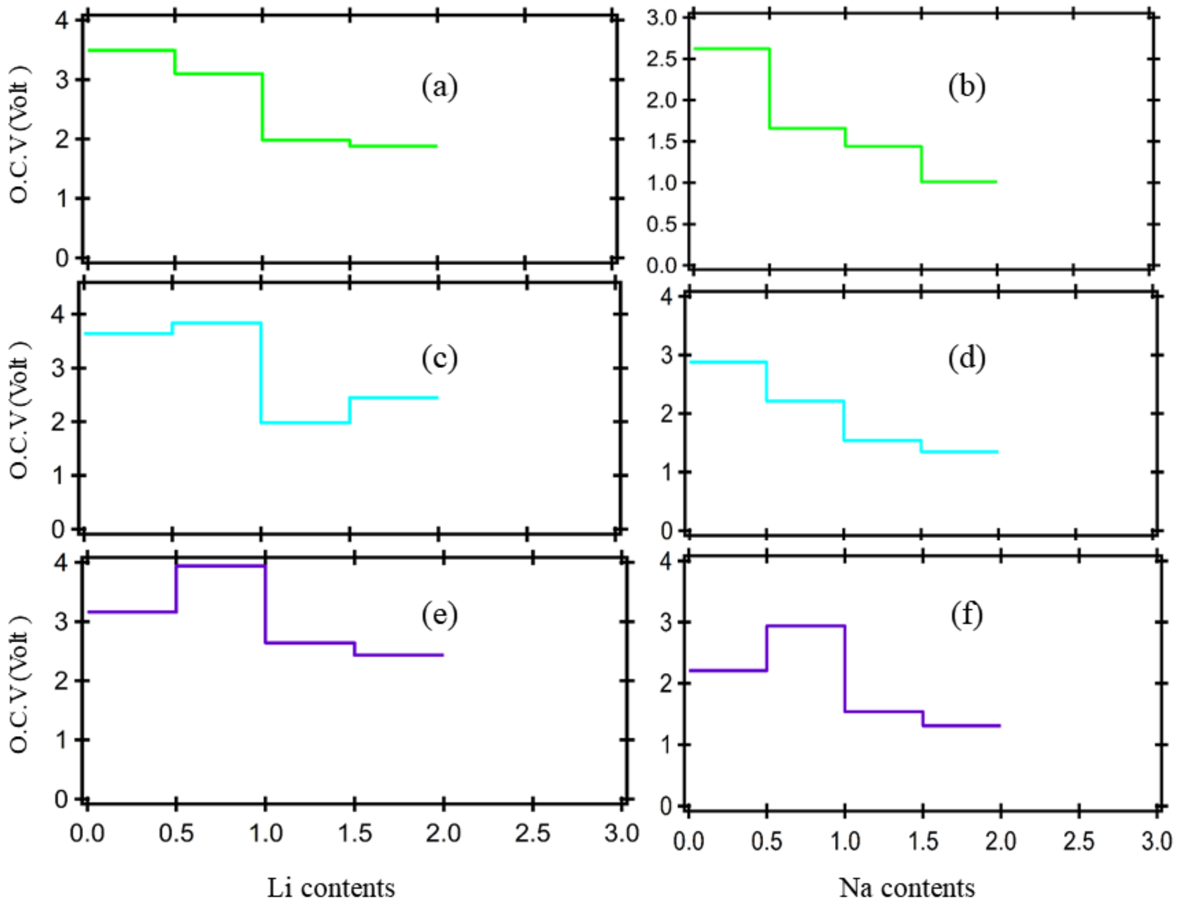


**Fig S9:** (a)/(b) O.C.V vs Li/Na contents of Ru doped $MnO_2$, (c)/(d) O.C.V vs Li/Na contents of Ru doped $CoO_2$, (e)/(f) O.C.V vs Li/Na contents of Ru doped $NiO_2$
